# Semen Collection and Evaluation in Two Tigers (*Panthera tigris*) and Two Leopards (*Panthera pardus*)

**DOI:** 10.3390/ani14162381

**Published:** 2024-08-16

**Authors:** Marco Cunto, Giulia Ballotta, Daniele Zambelli

**Affiliations:** Department of Veterinary Medical Science, University of Bologna, Ozzano dell’Emilia, 40064 Bologna, Italy; marco.cunto@unibo.it (M.C.); daniele.zambelli@unibo.it (D.Z.)

**Keywords:** wild felids, *Panthera tigris*, *Panthera pardus*, semen collection, artificial reproductive techniques, endangered species conservation

## Abstract

**Simple Summary:**

In this study, semen collection techniques, in particular, Urethral Catheterization after Pharmacological Induction (Ur.Ca.P.I.) and electroejaculation, have been applied in two tigers and two leopards. Information about sperm collection in tigers and leopards has been reported, describing the authors’ experience and presenting new data about a topic difficult to study due to the animal species involved. In particular, collection was possible in both species thanks to the use of electroejaculation. Ur.Ca.P.I. gave positive results in leopards, while in tigers, this technique did not allow satisfactory results.

**Abstract:**

Assisted reproduction techniques play a significant role in veterinary medicine, and although they are widely used in domestic animals, they are also becoming increasingly relevant in clinical practice for wild felids, especially in the conservation efforts for endangered species. In this study, the result of two semen collection techniques, urethral catheterization after pharmacological induction (Ur.Ca.P.I.) and electroejaculation, are described, aiming to provide new practical information about sperm collection using the Ur.Ca.P.I. technique and electroejaculation in tigers and leopards, describing the authors’ experience and presenting new data and observations. The following descriptive study included two subjects of *Panthera tigris* species and two of *Panthera pardus*. These subjects, after general anesthesia, underwent sperm collection initially with Ur.Ca.P.I. and, subsequently, with electroejaculation. Sampling was made possible in both species thanks to the use of electroejaculation. Sperm volumes in leopards ranged from 0.3 to 0.5 mL and in tigers from 0.5 to 2.177 mL. Sperm concentration in leopards ranged from 136 × 10^6^ to 280 × 10^6^ sperm/mL, and in tigers, from 21.5 × 10^6^ to 354 × 10^6^ sperm/mL. Urethral catheterization gave positive results in leopards, with sperm volumes ranging from 25 up to 150 µL and a concentration ranging from 110 × 10^6^ up to 1082 × 10^6^ sperm/mL. In tigers, unlike in leopards, the use of the Ur.Ca.P.I. technique encountered difficulties that did not allow satisfactory results to be obtained. Therefore, it would be useful to test the feasibility of urethral catheterization on a larger group of individuals in order to have more meaningful feedback. Finally, because electroejaculation always allowed semen collection in tigers, with a higher sperm quality than samples collected by Ur.Ca.P.I., we currently consider it the technique of choice for the collection of semen material in this species.

## 1. Introduction

All felid species (*Felidae*), except for the domestic cat, are at risk of extinction in their natural habitat. For this reason, the creation of captive populations of wild felids is considered useful both for the purpose of having a “reserve” of animals and, at the same time, for research opportunities that would otherwise not be available in the wild [1]. Artificial insemination and other assisted reproductive techniques, such as in vitro fertilization, embryo transfer as well as gamete collection and preservation techniques, are important tools for the management and preservation of wild felid populations and provide practical solutions to overcome mating difficulties encountered in captivity [2,3,4,5,6]. However, although these techniques have been used successfully in some felid species, success rates remain low [1,6]. In particular, several semen collection methods could be used in both domestic animals and different wild mammals [7]. Urethral catheterization after pharmacological induction (Ur.Ca.P.I.), the last semen collection technique described since 2008, is a simple and repeatable technique, initially described in the domestic cat, that exploits the α-adrenergic effect of some drugs commonly used for anesthesia [8]. Our research team, in the last few years, has wildly improved and used this technique in domestic cats [9,10,11,12]**,** and literature data report that Ur.Ca.P.I. in wild animals permits the collection of good results in the lion, the golden Asiatic cat, the caracal, and the jaguar, proving potentially applicable to other felids as well [7,13,14]. Data about sperm collection by Ur.Ca.P.I. and electroejaculation (EE) in small groups of animals have also been reported in different subspecies of leopards and tigers [15,16,17].

Information reported in the literature regarding EE and Ur.Ca.P.I. is scarce, especially concerning the Ur.Ca.P.I. technique, first described in the domestic cat [8] and applied by some authors only on a few wild felids [7]. Thus, considering the few animals on which these techniques have been applied and the few data reported in the literature, the purpose of this study was to provide new practical information about sperm collection using the Ur.Ca.P.I. technique and EE in tigers and leopards, describing the authors’ experience and presenting new data and observations, improving knowledge on this topic.

Data were collected during visits made to the Permanent Wildlife Exhibition “Tiger Experience” to evaluate males for which the owner intended to schedule natural matings or artificial inseminations. All semen collections were scheduled during routine control visits.

## 2. Materials and Methods

***Animals***

This study included male animals housed at the Permanent Wildlife Exhibition “Tiger Experience” in Campolongo Maggiore (VE), Italy. The animals’ semen was evaluated at the request of the facility. Specifically, two tigers (*Panthera tigris*) and two leopards (*Panthera pardus*) were evaluated. The study was conducted by the Animal Reproduction Unit of the Department of Veterinary Medical Sciences at the University of Bologna in the period February–June. All procedures performed in this study were approved by the Ethics Committee of the University of Bologna (Prot. n. 211211/2024).

No alterations in the general condition of all subjects were reported in the history, and each of them appeared to be in good health at the time of semen collection.

Leopard 1 was 14 years old and weighed approximately 50 kg. The subject underwent sampling and evaluation of semen to perform artificial insemination, as he exhibited aggressive behavior toward the female despite the female showing oestrus signs.

Leopard 2 was 9 years old and weighed approximately 60 kg. Semen was collected and assessed because of suspected infertility, as no pregnancies were obtained after some matings with a fertile female.

Tiger 1 was 10 years old and weighed about 200 kg. The subject underwent evaluation of semen material in anticipation of artificial insemination because, although he had a normal libido and showed interest in the female, he was unable to complete mating because he carried himself too cranially during mating, reaching all the way over the female’s back instead of laying semen in the genital tract.

Tiger 2 was 6 years old and weighed about 180 kg. The subject underwent an evaluation of semen material, also in anticipation of artificial insemination, since it did not show interest in the female and consequently did not engage in copulation.

***Sperm collection***

The semen collection techniques used were Urethral Catheterization after Pharmacological Induction (Ur.Ca.P.I.) and electroejaculation (EE). Since Ur.Ca.P.I. has not yet been widely used in wild felids, it was performed in part based on the data proposed by Lueders et al. [16].

Semen collected was evaluated, first in the field and later also at the Reproduction Unit Laboratory of the Department of Veterinary Medical Sciences of the University of Bologna, where a more in-depth analysis was performed. The animals were evaluated several times throughout the year, with varying time lapses between collections, in conjunction with the needs of the requesting facility.

For each individual, multiple sessions were conducted for semen sampling, in each of which multiple sampling attempts were performed:

- Two sessions with a 2-month interval between them for Ur.Ca.P.I. and 1 session for electroejaculation, performed 1 month after the last Ur.Ca.P.I. session in leopard 1;
- Two sessions for Ur.Ca.P.I. with a 1-month interval between them and 1 for electroejaculation performed 1 month after the last Ur.Ca.P.I. session in leopard 2;
- Three sessions with a 1-month interval between them for Ur.Ca.P.I. and 1 for electroejaculation performed 1 month after the last Ur.Ca.P.I. session in tiger 1;
- Four sessions with a 1-month interval between them for Ur.Ca.P.I. and 1 for electroejaculation performed 1 month after the last Ur.Ca.P.I. session in tiger 2.

***Anesthesia***

The leopards were anesthetized using 50 μg/kg of medetomidine (Sedator^®^, Eurovet Animal Health BV, Bladel, NL, The Netherlands) in combination with 2.5 mg/kg of ketamine (Ketavet 100^®^, Intervet Productions S.r.l, Aprilia, LT, Italy), both administered intramuscularly (IM). Scoop-launched darts were used for administration, and when necessary, administration was supplemented with the use of a rod to achieve the appropriate anesthesia plane. In one case, the above doses were not sufficient to induce anesthesia, so it was necessary to use a total of 70 μg/kg medetomidine and 3 mg/kg ketamine.

The anesthesia procedure in tigers was performed similarly to the leopard but using 45 μg/kg of medetomidine and 1 mg/kg of ketamine.

At the end of each semen collection, atipemazole (Atipam^®^, Eurovet Animal Health BV, Bladel, NL, The Netherlands), at the dose of 0.15 IM mg/kg in the leopards and 0.1 mg/kg IM in the tigers, was administered to all subjects to antagonize α2-agonist effect and speed up awakening.

***Urethral catheterization after pharmacological induction (Ur.Ca.P.I.)***

For semen collection with Ur.Ca.P.I., different types of urinary catheters were used for sampling:

- Rigid dog catheter (Dog Catheter with Female Luer Mount) 50 cm long with 2 mm (6F) diameter;
- Rigid dog catheter (Dog Catheter with Female Luer Mount) 50 cm long with 2.6 mm (8F) diameter;
- Rigid dog catheter (Dog Catheter with Female Luer Mount) 60 cm long with 3.3 mm (10F) diameter.

Ur.Ca.P.I. was performed as described by Zambelli et al. [8]. Each catheter was previously cut with a scalpel blade at its terminal end for about 2 cm in order to eliminate the lateral openings characteristic of the urinary catheter. At the beginning of the urethral catheterization procedure, the catheter was lubricated with non-spermicidal gel so as to facilitate its entry into the urethra. The depth at which the catheter was introduced into the urethra in order to reach the prostate and collect semen by capillarity was measured starting from the tip of the everted penis to the cranial edge of the thigh, a point that corresponds to the physiological position of the prostate. Through this assessment, the measurements obtained were 23 cm for leopards and 28 cm for tigers. Thus, the catheter was inserted into the urethra and left in place for a few seconds to allow the seminal material to pass inside the lumen by capillarity. After that, the catheter was pulled out to observe its effective filling.

***Electroejaculation (EE)***

**Figure 1:**
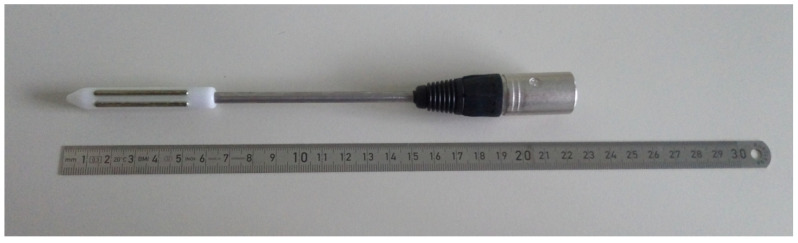
Electroejaculator probe. Total length 22 cm, portion with two electrodes length 6 cm and with a diameter of 1.27 cm.

Electroejaculation was performed using the same anesthesiological protocol used for Ur.Ca.P.I. A cat and dog probe with two electrodes (Figure 1) connected to an e320 Minitube^®^ electroejaculator (Minitube, Tiefenbach, Germany) was used for the procedure.

The proper position and depth of the probe within the rectum for adequate stimulation and subsequent ejaculation were assessed by the presence of the extensor movements of the hind limbs, characteristic of electroejaculation.

On the basis of that, in the two leopards, the probe was inserted at a depth of 14 cm. In the tigers, the same probe was used but applied a rigid extension around the cable, which allowed a greater depth within the rectum because of the difference in size between the two species. So, in the two tigers, the extension permitter to insert the probe at a depth of 24 cm.

The stimuli administered in both species were a total of 80 divided into 3 series (30, 30, 20): the first series of 10 stimuli at 3 V, 10 at 4 V, and 10 at 5 V; the second series of 10 stimuli at 4 V, 10 at 5 V and 10 at 6 V, and the last series of 10 stimuli at 5 V and 10 at 6 V, as described by Fukui et al. [17].

In both leopards and in tiger 2, electroejaculation was repeated a second time, five minutes apart from the first, using the same parameters.

***Sperm evaluation***

**Figure 2:**
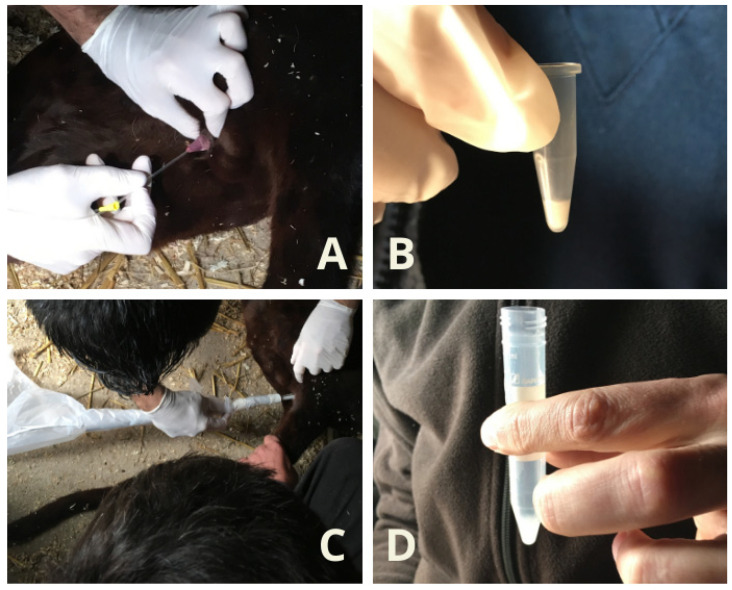
Semen collection in Leopard. (**A**) Ur.Ca.P.I. technique; (**B**) semen collected via Ur.Ca.P.I. transferred into a 1.5 mL Eppendorf; (**C**) electroejaculation technique; (**D**) semen collected via Ur.Ca.P.I. transferred into a 5 mL tube that had been previously heated.

**Figure 3:**
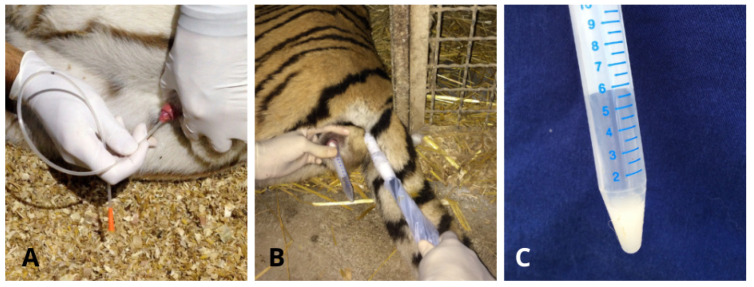
Semen collection in Tiger. (**A**) Ur.Ca.P.I. technique; (**B**) electroejaculation technique; (**C**) semen collected via Ur.Ca.P.I. transferred into a 5 mL tube that had been previously heated.

The semen collected using the Ur.Ca.P.I. technique was transferred in a 1.5 mL Eppendorf while the samples collected using the electroejaculation technique were put in a 5 mL tube that had been previously heated (Figure 2 and Figure 3). Semen collected by the two techniques was always first macroscopically evaluated on site (color and volume using a variable volume pipette), followed by microscopic evaluation, performed using a volume of 2–4 μL semen sample. In cases where the sperm concentration was very high, for a correct evaluation, samples were diluted 1:2 with TRIS, as described by Rota et al. [18]. Sperm motility and movement quality evaluation were performed using a phase-contrast microscope (Zeiss^®^ microscopes, Carl Zeiss S.p.A, Milato, Italy) with 400× magnification as described by Wildt et al. [15]. Then, viability was assessed with eosin-nigrosin staining, as described by Feldman and Nelson [19], and the percentage of normal spermatozoa and pathological forms was established by Rose Bengal- Fast Green FCF staining, as described by Pope et al. [20]. Semen concentration and total number of spermatozoa were calculated using a Burker chamber. The evaluation was concluded by observation at 400×/1000× of the field-stained slides using a Nikon^®^ Eclipse microscope (Nikon Europe B.V., Amstelveen, The Netherlands). For each slide, 200 spermatozoa were counted [21]. The incidence of spermatozoal malformations was calculated using the teratozoospermia index (TZI) described by Fabbris and Valiani [21]:

TZI = TOTAL ABNORMALITIES/ABNORMAL SPERMATOZOA.

## 3. Results

In leopards, Ur.Ca.P.I. was performed twice on different days by inserting a 6F catheter to a depth of 23 cm in both cases and animals. In tigers, the Ur.Ca.P.I. technique, applied by inserting the catheter to a depth of 28 cm (as hypothesized by measuring the tip of the penis-cranial margin of the thigh), did not permit semen collection. Therefore, different attempts were made to introduce the catheter deeper, trying to collect semen. In particular, in tiger 1, collection attempts were divided into the following:

Day 1:

- 10F catheter and 28 cm depth: no sample collected;
- 10F catheter and depth of 30 cm: 8 μL of semen collected; found an obstacle to continuing the insertion of the catheter;
- 10F catheter and depth of 40 cm: collected urine with spermatozoa.

Day 2:

- 8F catheter and depth of 28 cm: no sample collected;
- 8F catheter and depth of 30 cm: 25 μL sampled; found an obstacle to continuing the insertion of the catheter.

Day 3:

- 8F catheter and depth of 30 cm: no sample collected, and found an obstacle to continuing the insertion of the catheter;
- 6F catheter and depth of 30 cm: no sample collected; found an obstacle to continuing the insertion of the catheter.

In tiger 2, collection attempts were divided into the following:

Day 1:

- 10F catheter and depth of 28 cm: no sample collected;
- 10F catheter and depth of 30 cm: collected 5 μL of semen; found an obstacle to continuing the insertion of the catheter.

Day 2:

- 10F catheter and depth of 30 cm: no sample collected, found an obstacle to continuing the insertion of the catheter;
- 10F catheter and depth of 33 cm: collected urine with spermatozoa.

Day 3:

- 8F catheter and depth of 30 cm: no sample collected, found an obstacle to continuing the insertion of the catheter;
- 8F catheter and depth of 35 cm: collected urine with spermatozoa.

Day 4:

- 8F catheter and depth of 30 cm: collected 4 μL of semen; found an obstacle to continuing the insertion of the catheter;
- 8F catheter and depth of 30 cm: no sample collected; found an obstacle to continuing the insertion of the catheter.

In the two leopards, it was possible to collect seminal material with Ur.Ca.P.I. in all sessions, resulting in a volume of 25 μL with a concentration of 1082 × 10^6^ spermatozoa/mL, 150 μL with a concentration of 110 × 10^6^ spermatozoa/mL in leopard 1, and a volume of 42 μL with a concentration of 978 × 10^6^ spermatozoa/mL and 55 μL with a concentration of 845 × 10^6^ spermatozoa/mL in leopard 2.

In tiger 1, Ur.Ca.P.I. allowed semen collection only two times, but in both cases resulted in an absence of spermatozoa and the presence of only urethral cells.

In tiger 2, Ur.Ca.P.I. resulted in semen collection only in two cases by inserting the catheter into the urethra at 30 cm: in the first case, 5 μL were collected with a concentration of 90.5 × 10^6^ spermatozoa/mL, while in the second case, a volume of 4 μL with a concentration of 1096 × 10^6^ spermatozoa/mL was obtained.

Although it was possible to collect seminal material in tigers without the presence of urine, there was still an obstacle at a depth of about 30 cm that very often prevented the urinary bladder from being reached.

Data about sperm collected by urethral catheterization are summarized in Table 1.

Sperm volumes collected by EE in all animals range from 0.3 up to 2.177 mL with a minimum concentration of 21.5 × 10^6^ up to 354 × 10^6^ sperm/mL. Specifically, in both leopards, two EE sessions were performed, and sperm collection was always possible. The second session was performed 10 min after the first one. In particular, in leopard 1, 0.3 mL of semen with a concentration of 266 × 10^6^ spermatozoa/mL and 0.4 mL of semen with a concentration of 136 × 10^6^ spermatozoa/mL were respectively collected on session 1 and session 2. In leopard 2, on session 1, 0.5 mL of semen with a concentration of 280 × 10^6^ spermatozoa/mL was collected while, on session 2, EE permitted collection of 0.3 mL of semen with a concentration of 181 × 10^6^ spermatozoa/mL. Electroejaculation was performed one time in tiger 1, permitting the collection of 0.5 mL of semen with a concentration of 354 × 10^6^ spermatozoa/mL. In tiger 2, two EE sessions were performed, and the second session was performed 10 min after the first one. The two sessions allowed, respectively, the production of 0.562 mL of semen with a concentration of 107.34 × 10^6^ spermatozoa/mL and the production of 2.177 mL with a concentration of 21.5 × 10^6^ spermatozoa/mL.

Data about sperm collected by EE are summarized in Table 2.

In the tigers, spermatozoa collected, with the exception of those found in the urine, show a movement quality ranging from 2 to 4 and a percentage of motility from 10% to 60%, while in leopards, all samples show spermatozoa with movement quality of 4 and motility between 30% and 60%. The percentages of pathological spermatozoa ranged from 56% to 63%, and the teratozoospermia index (TZI) ranged from 1.23 to 1.39 in tigers, while in leopards, the percentages of pathological spermatozoa ranged from 61% to 75%, and the teratozoospermia index (TZI) ranged from 1.32 to 1.43. Because of the small volume, it was not possible to assess viability in all samples obtained (two leopard samples and one tiger sample); in all samples collected, this parameter never exceeds 62%; in particular, viability in tigers ranged from 34% to 62% and in leopards from 35% to 47%. The results are reported, according to the sampling method used, in Table 1 and Table 2.

## 4. Discussion

Urethral catheterization in the two leopards has been successfully performed as described by Lueders et al. [16]**,** although the catheter insertion depth that allowed collection was less than that reported in his study (23 cm instead of 30). The authors hypothesize that the animal used in Lueders et al.’s study [15] was smaller in size compared to the two leopards enrolled in this study. Except for this detail, semen collected is characterized by a low volume and high concentration of spermatozoa as reported in the African leopard [16] and similar to what has been described in the domestic cat [22]. In contrast, electroejaculation allowed the collection of a greater volume of sperm than Ur.Ca.P.I. due to the greater production of seminal plasma by the accessory sex glands using this method, as occurs during electroejaculation in the domestic cat [23]. Nevertheless, the concentration of semen collected by EE appears to be higher than that reported by Wildt et al. in the leopard, although the number of total spermatozoa is smaller because the volume is smaller [15]. Considering the small group of animals in both papers (*n* = 2 in this study and *n* = 4 in the study by Wildt et al. [15]), this difference is probably due to individual aspects or, alternatively, could be related to the different subspecies of leopard tested in the studies.

Concerning the two tigers, Ur.Ca.P.I. was successful only in tiger 2 and only two times out of eight attempts. In one of these, both the volume and the concentration of semen obtained did not reach the expectations proposed by this technique. The other successful catheterization in tigers had a high concentration but an extremely small volume compared to that collected by Lueders et al. [16]. Furthermore, on both occasions that allowed sperm collection, the presence of resistance near the 30 cm depth was detected.

In tiger 1, Ur.Ca.P.I. did not permit sperm collection, except for two occasions where samples collected were without spermatozoa.

In both tigers, the catheter frequently did not advance into the urethra beyond 30 cm in depth, even when using a smaller diameter catheter, due to a physical impediment near the prostate (located approximately at the cranial margin of the thigh, as an external reference). This impediment prevents adequate and smooth progression of the catheter and, consequently, sperm collection. However, this obstacle was not reported during sperm collection performed by Lueders et al. using Ur.Ca.P.I. [16]. We hypothesize that this difficulty in catheter progression could be due to a particular conformation of the urethra (such as a possible narrowing) or to the presence of openings of some ducts (e.g., deferens on the seminal colliculus), where the catheter could be blocked, or, again, to the presence of flexures of the urethra. The latter could be induced by the subject’s position in decubitus during semen collection procedure, by bladder repletion, or an anatomical feature of the tiger in general or of the subspecies used in this study. Despite the presence of this obstacle, it was possible to pass 30 cm once in tiger 1 and twice in tiger 2. In these cases, the catheter reached the urinary bladder, and on all these occasions, urine samples containing a moderate amount of sperm were collected. This indicates the production of sperm in both tigers, but probably the Ur.Ca.P.I., while allowing their release in the urethra, makes it difficult to retrieve them except in the bladder. The authors suppose that this aspect could also play a role, considering the poor quality of sperm collected in one out of two attempts in tiger 2. In particular, samples collected were inferior in quality and quantity compared to the subject’s potential, as evidenced by sperm collected by EE, which presented an ejaculate within the normal range. The failure or reduction of sperm collected by Ur.Ca.P.I., even if the estimated position of the prostate was reached, may be due to the previously hypothesized particular urethral conformation that induces partial or total passage of sperm into the urinary bladder during contraction of the vas deferens induced by medetomidine. This can be supported by the presence of spermatozoa observed in urine whenever it was possible to advance the catheter deeper than 30 cm into the urethra, although finding a moderate amount of sperm in the urinary bladder is considered a normal phenomenon in domestic cats and other species [24].

Similarly to the leopard, EE allowed sperm collection whenever it was performed on the tigers, and although the volume was lower than in the tigers evaluated by Wildt et al. and Fukui et al. [15,17], the concentration of spermatozoa per ml was found to be higher. This could be due to some differences in electrical stimuli provided during the EE procedure and/or to differences in the anesthetic protocol used by various authors that could have influenced sperm collection. In fact, Wildt et al. [15] administered only ketamine to perform EE, and it has been shown, in domestic cats, that the use of medetomidine respects the administration of only ketamine-influenced concentration of sperm collected, resulting in higher samples collected after medetomidine administration [24]. It is possible to suppose a similar influence also in tigers and other felids. Again, different subspecies tested or animals’ individual features or conditions could underlie the observed differences, considering the small number of subjects tested in all papers published about sperm collection in wild felids.

Repeating the electroejaculation 10 min after the first ejaculation results in lower concentration in both leopards and tiger 2 samples than in the first ejaculate. These results are in line with our expectations.

Although a type 4 quality movement (moderate and constant progressive movement) characterized spermatozoa in all samples obtained, except for the one collected by Ur.Ca.P.I. in tiger 1 (movement 2), the highest motility was observed with the use of EE, where the percentage reaches 60% in both species. This aspect, already observed also in the domestic cat (Zambelli, personal observation) probably can be attributed to the scarcity of seminal plasma in Ur.Ca.P.I. samples. In fact, in our experience, sperm motility usually improves after dilution with a TRIS extender.

**Figure 4:**
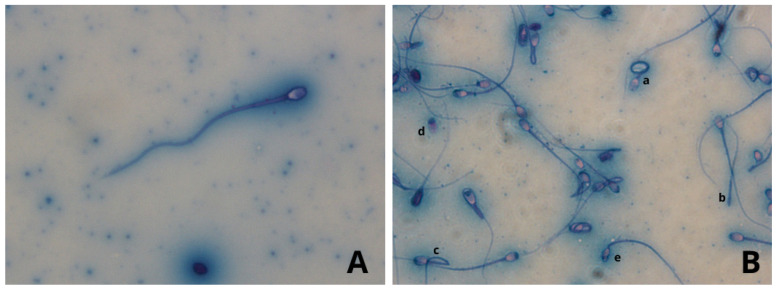
Leopard seminal material with Rose Bengal Fast Green stain. Stage photographs were taken at 400× magnification. (**A**) normal spermatozoa. (**B**) most commonly found malformation, a: twisted tails, b: detached tail, c: bent tail, d: detached head, e: microcephalus.

**Figure 5:**
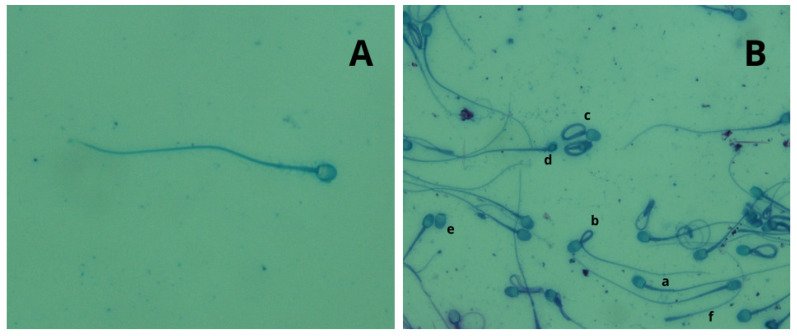
Tiger seminal material with Rose Bengal Fast Green stain. Stage photographs were taken at 400× magnification. (**A**) normal spermatozoa. (**B**) normal (a) and malformed spermatozoa, b: bent tail, c: twisted tails, d: microcephalus, e: detached head, f: detached tail.

Regardless of the collection technique used, all semen samples collected from both species were characterized by a high percentage of abnormal spermatozoa, confirming the high degree of teratospermia that characterizes most feline species, as previously reported by Pukazhenthi et al. [25]. The percentage of altered spermatozoa (teratospermia) was high in all subjects and in all sperm samples collected both by Ur.Ca.P.I. and by EE. Teratospermia was most evident in the leopards, where altered spermatozoa reach up to 75%, a value rather similar to that described by Wildt et al. [15], who reported 80% of spermatozoa with malformations. Even if, in this study, teratospermia was higher in leopards than in tigers, the latter exhibited abnormalities with a percentage never below 56%, a value significantly higher compared with the data reported by Wildt et al. (37.5%), Donoghue et al. (8–10%) and Pukazhenthi et al. (40%) [15,26,27]. On the basis of these results, we cannot exclude that ejaculatory abstinence or the presence of disorders inducing teratospermia were the basis of these observations in tigers tested in our study. The teratozoospermia index showed that among the malformed sperms, there are alterations in several sperm traits in all samples taken, particularly in leopards, where a TZI of 1.43 was reached. The most commonly found malformations were in the head and tail, with acrosomal abnormalities, detached heads, elongated heads, twisted tails, and bent tails predominating (Figure 4 and Figure 5). The types of spermatozoa defects observed were not unusual based on previously detailed analyses in other Felidae [15].

## 5. Conclusions

In conclusion, this study presents some limitations, in particular, due to the low number of animals involved and the consequent impossibility of performing a statistical comparison of the results obtained. Furthermore, performing sperm collection procedures in the field reduced the possibility of analyzing more in-depth semen samples collected as it would have been possible and appropriate to do in a dedicated laboratory. However, this paper provides very important information about sperm collection in tigers and leopards, describing the authors’ experience and presenting new data and observations about a topic difficult to study due to the animal species involved. Even if referred to a small number of animals, our results confirmed that Ur.Ca.P.I. is an easily feasible, repeatable, and inexpensive technique for collecting semen in leopards, as reported by Lueders et al. [16], similar to what was reported in the domestic cat [22], and it can be considered a good alternative to EE. The latter can still be considered an effective collection technique for the leopard, although it requires the use of specific and more expensive equipment [7]. While in the tiger, in our experience, although Ur.Ca.P.I. is executable, it was difficult to perform due to a frequent physical impediment at 30 cm in depth, which often does not permit the advancement of the catheter in the urethra to the prostate and, consequently, the collection of semen. This finding has never been reported previously in the literature; therefore, it would be useful to test the feasibility of urethral catheterization on a larger group of individuals in order to have more meaningful feedback about this aspect. In addition to this, investigations with imaging methods such as ultrasonography and/or ascending urography with contrast medium should be studied to assess the true conformation of the urethra and to verify the presence and nature of this impediment. Ultrasonography could also allow guided catheterization to be performed. Therefore, based on the results obtained in this study, we currently consider Ur.Ca.P.I. to need further investigation and it is evidently not usable for sperm collection in all tigers. In contrast to Ur.Ca.P.I., EE always allowed semen collection in tigers, with a higher sperm quality with respect to the samples collected by Ur.Ca.P.I. Therefore, we currently consider it the technique of choice for the collection of semen material in this species.

## Figures and Tables

**Table 1:** Sample collected via the Ur.Ca.P.I. method.

Animal	Day	DepthCatheter Insertion [cm]	Sample Collected	Volume [μL]	Concentration [SPTZ ^2^/mL]	Total SPTZ ^2^ [×10^6^]	Motility [%]	Movement	Pathological SPTZ ^2^ [%]	Viability [%]	TZI ^3^
Leopard 1	1	23	Semen	25	1082 × 10^6^	27.05	30	4	67	35	1.32
	2	23	Semen	150	110 × 10^6^	16.5	30	4	70	47	1.35
Leopard 2	1	23	Semen	42	978 × 10^6^	41.07	40	4	68	41	1.33
	2	23	Semen	55	845 × 10^6^	46.47	30	4	69	38	1.34
Tiger 1	1	28 30 40	/ Secretion Urine with SPTZ ^2^	/ 8 NV ^1^	/ No SPTZ ^2^ NV ^1^	/ No SPTZ ^2^ NV ^1^	/ / /	/ / /	/ / /	/ / /	/ / /
	2	28 30	/ Secretion	/ 25	/ No SPTZ ^2^	/ No SPTZ ^2^	/ /	/ /	/ /	/ /	/ /
	3	30 30	/ /	/ /	/ /	/ /	/ /	/ /	/ /	/ /	/ /
Tiger 2	1	28 30	/ Semen	/ 5	/ 90.5 × 10^6^	/ 452.5	/ 10	/ 2	/ 56	/ 34	/ 1.23
	2	30 33	/ Urine with SPTZ ^2^	/ NV ^1^	/ NV ^1^	/ NV ^1^	/ /	/ /	/ /	/ /	/ /
	3	30 35	/ Urine with SPTZ ^2^	/ NV ^1^	/ NV ^1^	/ NV ^1^	/ /	/ /	/ /	/ /	/ /
	4	30 30	Semen /	4 /	1096 × 10^6^ /	4.384 /	50 /	4 /	63 /	52 /	1.39 /

^1^ NV: Not valuated, ^2^ SPTZ: Spermatozoa, ^3^ TZI: Teratozoospermia index.

**Table 2:** Sample collected via electroejaculation method.

Animal	Depth Catheter Insertion [cm]	Sample Collected	Volume [mL]	Concentration [SPTZ ^1^/mL]	Total SPTZ ^1^ [×10^6^]	Motility [%]	Movement	Pathological SPTZ ^1^ [%]	Viability [%]	TZI ^2^
Leopard 1	14	Semen	0.4 0.3	136 × 10^6^ 266 × 10^6^	54.4 79.8	60 60	4 4	75 61	NV ^3^ NV ^3^	1.36 1.43
Leopard 2	14	Semen	0.5 0.3	280 × 10^6^ 181 × 10^6^	140 54.3	60 30	4 4	63 76	40 36	1.43 1.43
Tiger 1	24	Semen	0.5	354 × 10^6^	177	50	4	62	53	1.33
Tiger 2	24	Semen	0.562 2.177	191 × 10^6^ 21.5 × 10^6^	107.34 46.8	60 60	4 4	59 57	NV ^3^ 62	1.38 1.29

^1^ SPTZ: Spermatozoa, ^2^ TZI: Teratozoospermia index, ^3^ NV: Not evaluated.

## Data Availability

The data presented in this study are available on request from the corresponding author.
